# Inductively Coupled Plasma Mass Spectrometry Performance for the Measurement of Key Serum Minerals: A Comparative Study With Standard Quantification Methods

**DOI:** 10.1002/jcla.25140

**Published:** 2024-12-23

**Authors:** Kosuke Kojo, Tomoko Oguri, Takazo Tanaka, Atsushi Ikeda, Takuya Shimizu, Shunsuke Fujimoto, Ayumi Nakazono, Yoshiyuki Nagumo, Shuya Kandori, Hiromitsu Negoro, Hiroyuki Nishiyama

**Affiliations:** ^1^ Department of Urology Institute of Medicine University of Tsukuba Tsukuba Japan; ^2^ Center for Human Reproduction International University of Health and Welfare Hospital Nasushiobara Japan; ^3^ Research Institute of Science for Safety and Sustainability (RISS) National Institute of Advanced Industrial Science and Technology (AIST) Tsukuba Japan; ^4^ Health Care Analysis Center Renatech Co. Ltd. Isehara Japan

**Keywords:** analytical performance, clinical biochemistry, essential mineral quantification, hemolysis, ICP‐MS, method comparison, outlier detection, serum trace elements, surplus or deficiency, total phosphorus

## Abstract

**Background:**

Inductively coupled plasma mass spectrometry (ICP‐MS) is widely used for the accurate measurement of minerals. However, its application to serum essential mineral measurement has not been fully evaluated. The present study aimed to assess the performance of ICP‐MS for serum minerals by comparing its measurements to those obtained using standard quantification methods.

**Methods:**

Cross‐sectional data were collected from 282 participants from a single facility in Japan. Serum concentrations of eight key minerals, namely sodium, potassium, calcium, phosphorus, magnesium, iron, zinc, and copper, measured via ICP‐MS and standard methods were compared using Passing–Bablok regression and Bland–Altman plots.

**Results:**

All minerals, except phosphorus, exhibited good agreement with standard methods, with more stable regression coefficients observed for minerals with greater interindividual variability. After systematically filtering outliers, the mean relative errors were approximately −3% for sodium, potassium, calcium, and magnesium; +5% for iron; 0% for zinc; and −19% for copper. The outliers for iron were primarily due to mild hemolysis, whereas those for zinc were largely attributed to nonhemolysis factors. For phosphorus, the serum total phosphorus concentration measured using ICP‐MS was approximately 3.5 times higher than the serum inorganic phosphorus concentration measured using standard methods, with a weak correlation observed between the two methods.

**Conclusion:**

This study provides a practical foundation for future research. Understanding ICP‐MS characteristics will facilitate the development of new approaches in clinical diagnostics.

## Graphical abstract

Analysis of real‐world cross‐sectional study data revealed relative errors between the standard method and ICP‐MS for the different minerals tested. Additionally, several outliers were observed exclusively in the ICP‐MS results, likely due to hemolysis or other unidentified factors. Although these limitations in ICP‐MS performance cannot be entirely dismissed, comparative results of the standard method and the ICP‐MS approach exhibited good agreement. The unique characteristics of ICP‐MS identified in this study lay a strong foundation for future research, not only for routine clinical measurement of specific minerals but also for disease‐specific analyses leveraging the ability of ICP‐MS to simultaneously measure a wide range of parameters.

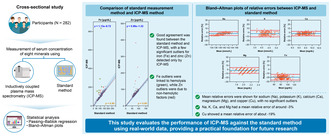

## 1 Introduction

Minerals are essential nutrients crucial for homeostasis. Certain minerals, such as sodium, chlorine, potassium, calcium, phosphorus, and magnesium, are required in amounts exceeding 100 mg/day, whereas others, such as iron, zinc, copper, selenium, and iodine, are needed in lower quantities [1]. All these minerals are considered fundamental nutrients for human health. Additionally, despite being present in trace amounts, manganese, chromium, and molybdenum, are essential for life [2]. Notably, Japanese dietary reference intakes for these minerals have been established by the Ministry of Health, Labour and Welfare of Japan [3]. Moreover, in Japan, sodium, chlorine, potassium, calcium, phosphorus, magnesium, iron, zinc, and copper are the clinically most focused minerals [4], given the relatively low incidence of deficiencies in selenium, iodine, manganese, chromium, and molybdenum within the standard Japanese diet [3]. Therefore, these nine minerals are predominantly measured in Japan, given their clinical significance and requirement for optimal health.

The comprehensive and simultaneous analysis of multiple minerals represents a promising approach in diagnostics [5, 6]. While single‐mineral measurements are important for rare conditions, such as Gitelman syndrome [7], hemochromatosis [8], and Wilson's disease [9], they have limited public health impact. Although not yet standard practice, advanced multimineral analysis techniques can offer potential insights into mineral dynamics and may reveal imbalances associated with diseases, such as Alzheimer's, Parkinson's, cardiovascular diseases, cancer, and diabetes [10, 11]. Among these techniques, inductively coupled plasma mass spectrometry (ICP‐MS), proposed since the 1980s [12], is a leading technique for simultaneously measuring multiple minerals. This technique offers high sensitivity, low detection limits, a wide dynamic range, and the ability to detect any element in the periodic table that can be ionized into positively charged ions [13]. ICP‐MS can simultaneously quantify major elements, such as sodium, and trace elements, such as selenium and manganese, using only 100 μL serum sample, offering benefit for pediatric patients, from whom sample collection is challenging [14, 15].

Despite the technological establishment of ICP‐MS, its widespread use in clinical settings remains limited owing to high equipment and operational costs and the need for highly specialized manpower [16]. The use of ICP‐MS has surpassed that of atomic absorption spectrometry over the last 25 years; however, it remains limited to monitoring trace minerals, such as zinc and copper, and toxic substances, such as arsenic and cadmium [16]. Although the accuracy of ICP‐MS is ensured with the use of standard reference materials, its performance in real‐world blood samples remains underexplored [17]. Moreover, the discrepancies between standard materials and clinical samples, due to complex matrices and interferences, underscore the need to evaluate ICP‐MS in practical clinical settings for reliable routine diagnostics [17].

A similar technique for measuring minerals in serum is inductively coupled plasma optical emission spectroscopy (ICP‐OES), also known as inductively coupled plasma atomic emission spectroscopy (ICP‐AES), which has been used since the 1970s [18]. ICP‐OES, with detection limits in the parts per billion range as compared to the parts per trillion achievable with ICP‐MS, has been also commonly used for substances that do not require such high sensitivity [19]. In clinical research, even for studies measuring multiple minerals simultaneously, ICP‐OES is commonly used for relatively high‐concentration minerals, whereas ICP‐MS is employed primarily for trace minerals, often resulting in the combined use of both methods [20]. However, comprehensive studies using ICP‐MS as the sole method to measure both relatively high‐concentration and trace minerals are comparatively rare. Thus, the performance of ICP‐MS in evaluating simultaneously both types of minerals, especially including relatively high‐concentration minerals, using a single technique remains insufficiently explored.

We have previously established a database from a cross‐sectional study that includes real‐world blood sample data and newly acquired ICP‐MS measurements [21]. This database includes information related to serum concentrations of sodium, potassium, calcium, phosphorus, magnesium, iron, zinc, and copper measured using standard quantification methods. In the present study, we aimed to evaluate the performance of ICP‐MS measurements of these eight key minerals. Notably, while standard methods typically measure inorganic phosphorus [4, 22, 23, 24], ICP‐MS measures total phosphorus [25, 26, 27, 28], which can lead to significant differences even in the same serum sample. Given the lack of prior studies where both inorganic and total phosphorus were measured simultaneously, the present study also included a comparative evaluation of inorganic and total phosphorus concentrations. Furthermore, although it was initially assumed during the establishment of the database that there would be no differences in the measurement targets for minerals other than phosphorus between ICP‐MS and standard methods, we reviewed the relevant literature to validate this assumption.

## 2 Materials and Methods

### 2.1 Study Design

This study utilized a dataset from 299 participants who had been recruited in a cross‐sectional project aimed at developing new diagnostic strategies for male infertility [21]. The participants were men who presented for consultation about infertility at the International University of Health and Welfare Hospital (Tochigi, Japan) between August 2019 and April 2022. All participants had an Eastern Cooperative Oncology Group Performance Status of 0 [29], indicating that they were nearly healthy. In previous reports [21], participants with a history of cancer were excluded owing to the potential impact of cancer history on mineral profiles [30] and those suspected of azoospermia—cases where all semen samples were used to confirm the complete absence of sperm—were excluded owing to the lack of remaining semen samples available for ICP‐MS evaluation. However, in this study, all such participants were included to ensure participant physiological diversity as much as possible. Seventeen individuals were excluded from the study owing to incomplete blood examination data—such as cases where blood samples were not collected at the time of enrollment as the patients had undergone prior infertility testing at other hospitals—resulting in a final cohort of 282 participants with complete datasets.

Blood samples collected from the arm veins of the participants were promptly processed into serum using the method described below. These serum samples were used to assess serum mineral levels using standard methods in the hospital's laboratory as part of routine clinical practice. An additional portion of the blood sample was collected, processed into serum using the same method, and subsequently frozen for comprehensive analysis of trace elements using ICP‐MS. The results from the ICP‐MS analysis were then compared with those obtained from the standard methods.

### 2.2 Characteristics of Study Participants

The age distribution of the participants is presented in Table S1. The median age was 35 years (interquartile range [IQR], 32–41 years). Only eight participants (2.8%) were over 50 years old.

According to international guidelines [31], 161 participants (57.1%) had abnormal semen findings, while 121 participants (42.9%) were classified as normozoospermic. However, owing to the limitations in the predictive accuracy of semen analysis, the possibility that their fertility potential may be abnormal cannot be ruled out [20] (Figure S1).

### 2.3 Screening of Serum Minerals Using Standard Methods

The concentrations of eight serum minerals were measured using the widely implemented standard methods in Japan [4, 32, 33, 34, 35, 36, 37, 38] (Table 1). The most commonly used reference intervals in Japan were employed to determine excess or deficient serum concentrations [4, 39, 40, 41, 42] (Table 1).

**TABLE 1:** Mineral assays and reference intervals.

Minerals	Assay methods	Reagents	Source for original methods	RIs	Source for RIs
Sodium	ISE^a^	Not applicable	Smith [32]	138–145 mmol/L	C‐CRI and JCCLS [39]
Potassium	ISE^a^	Not applicable	Smith [32]	3.6–4.8 mmol/L	C‐CRI and JCCLS [39]
Calcium	Enzyme assay^a^	α‐amylase^b^	Kayamori [33]	8.8–10.1 mg/dL	C‐CRI and JCCLS [39]
Phosphorus	Enzyme assay^a^	PNPase and XDH^b^	Machida [34]	2.7–4.6 mg/dL	C‐CRI and JCCLS [39]
Magnesium	Enzyme assay^a^	ATP and hexokinase^b^	Tabata [35]	1.8–2.5 mg/dL	JCOG [40]
Iron	Colorimetric assay^a^	Nitroso‐PSAP^c^	Saito [36]	40–188 μg/dL	C‐CRI and JCCLS [39]
Zinc	Colorimetric assay^a^	5‐Br‐PAPS^d^	Makino [37]	80–130 μg/dL	JSCN [41]
Copper	Colorimetric assay^e^	3,5‐DiBr‐PAESA^f^	Abe [38]	71–132 μg/dL	Traditional [42]

Abbreviations: 5‐Br‐PAPS, 2‐(5‐bromo‐2‐pyridylazo)‐5‐[N‐propyl‐N‐(3‐sulfopropyl)amino]phenol; ATP, Adenosine triphosphate; C‐CRI, the Committee on Common Reference Intervals of the Japan Society of Clinical Chemistry; ISE, ion‐selective electrode; JCCLS, the Japanese Committee for Clinical Laboratory Standards; JCOG, the Japan Clinical Oncology Group; JSCN, the Japanese Society of Clinical Nutrition; Nitroso‐PSAP, 2‐nitroso‐5‐[N‐n‐propyl‐N‐(3‐sulfopropyl)amino]phenol; PNPase, polynucleotide phosphorylase; RI, reference interval; XDH, xanthine dehydrogenase; 3,5‐DiBr‐PAESA, 4‐(3,5‐dibromo‐2‐pyridylazo)‐N‐ethyl‐N‐(3‐sulfopropyl)aniline.

^a^ LABOSPECT 006 or 008 (Hitachi Hi‐Tech Do Ltd., Tokyo, Japan).

^b^ L type (FUJIFILM Wako Pure, Osaka, Japan).

^c^ Quick Auto Neo Fe (Shino‐Test, Tokyo, Japan).

^d^ Accuras Auto Zn (Shino‐Test, Tokyo, Japan).

^e^ JCA‐BM8020 (JEOL Ltd., Tokyo, Japan) at a contract laboratory (SRL Inc., Tokyo, Japan).

^f^ Quick Auto Neo Cu (Shino‐Test, Tokyo, Japan) at a contract laboratory (SRL Inc., Tokyo, Japan).

### 2.4 Measurement of Serum Minerals Using ICP‐MS

The dataset referenced in the cross‐sectional research of the present study focuses on measuring the concentrations of various minerals in serum, the liquid component of blood devoid of cellular components and coagulation factors, using ICP‐MS. For this analysis, 6 mL of blood was specifically collected for ICP‐MS analysis and processed into serum following the standard laboratory protocols. This procedure involved allowing the blood sample to clot at room temperature for over 30 min, followed by centrifugation at 1500× *g* for 10 min to isolate the supernatant [43]. The serum was stored at −80°C until subsequent measurements.

The concentrations were measured using ICP‐MS, following previously reported methods [21, 44, 45]. Detailed information about the ICP‐MS system is summarized in Table 2. The limits of quantification for each mineral were calculated prior to the study, following the definition established by the Japanese Industrial Standard [46], and are summarized in Table 2. ICP‐MS measurements were reported in mg/L or μg/L. To align with the standard units used in Japan, conversions were performed using the formulas presented in Table 2. Mass concentrations [mg/L] were converted to molar concentrations [mmol/L] by dividing by the molar mass [47]. Prior to the study, Seronorm Trace Elements Serum Level 1 (Sero, Billingstad, Norway) was analyzed using the same procedure for quality control, as detailed in Table S2.

**TABLE 2:** Summary of inductively coupled plasma mass spectrometry measurement conditions.

Item	Details
Measurement system	Agilent 7800 (Agilent Technologies, Santa Clara, CA, USA)
Sample preparation	50 μL of serum mixed with 125 μL of a 61% nitric acid solution and 25 μL of a 30% hydrogen peroxide solution, heated at 70°C for 16 h. An internal standard was added and diluted to 2.5 mL with ultrapure water.
Standard solutions	– Standard solutions^a^: sodium, potassium, calcium, and magnesium – Standard solutions (FUJIFILM Wako Pure Chemical Corporation, Osaka, Japan): phosphorus – XSTC‐622B (SPEX CertiPrep, Metuchen, NJ, USA): iron, zinc, copper
Measurement conditions	– RF power: 1550 W – Plasma gas flow rate: 15 L/min – Nebulizer gas flow rate: 1.05 mL/min – Collision/reaction gas flow rate: 6.0 mL/min (hydrogen gas), 4.3 mL/min (helium gas)
Calibration curves	Correlation coefficient for each mineral ≥ 0.9998 Concentration ranges for the calibration curves: – Sodium: 10, 50, and 100 mg/L – Potassium, Calcium, Phosphorus: 0.5, 2.5, and 5 mg/L – Magnesium: 0.1, 0.5, and 1 mg/L – Iron: 2, 10, 50, and 250 μL – Zinc and Copper: 2, 10, and 50 μL

**Analytical parameters for each mineral**	**Minerals**	**Internal standard elements** ^**a**^	**Collision gas**	**Limit of quantitation**	**Unit conversion formula**
	Sodium	Beryllium‐9	Helium gas	$1.61\times 10^{-2}\;\left[\mathrm{mg}/L\right]$	$y\left[\text{mmol}/L\right]=\frac{x\left[\mathrm{mg}/L\right]}{22.99}$
	Potassium	Yttrium‐89	Hydrogen gas	$2.73\times 10^{-3}\;\left[\mathrm{mg}/L\right]$	$y\left[\text{mmol}/L\right]=\frac{x\left[\mathrm{mg}/L\right]}{39.10}$
	Calcium	Yttrium‐89	Hydrogen gas	$1.00\times 10^{-2}\;\left[\mathrm{mg}/L\right]$	$y\left[\mathrm{mg}/\mathrm{dL}\right]=\frac{x\left[\mathrm{mg}/L\right]}{10}$
	Phosphorus	Beryllium‐9	Helium gas	$4.42\times 10^{-3}\;\left[\mathrm{mg}/L\right]$	$y\left[\mathrm{mg}/\mathrm{dL}\right]=\frac{x\left[\mathrm{mg}/L\right]}{10}$
	Magnesium	Beryllium‐9	Helium gas	$4.32\times 10^{-4}\;\left[\mathrm{mg}/L\right]$	$y\left[\mathrm{mg}/\mathrm{dL}\right]=\frac{x\left[\mathrm{mg}/L\right]}{10}$
	Iron	Yttrium‐89	Hydrogen gas	$0.789\;\left[\mathrm{\mu g}/L\right]$	$y\left[\mathrm{\mu g}/\mathrm{dL}\right]=\frac{x\left[\mathrm{\mu g}/L\right]}{10}$
	Zinc	Yttrium‐89	Helium gas	$0.496\;\left[\mathrm{\mu g}/L\right]$	$y\left[\mathrm{\mu g}/\mathrm{dL}\right]=\frac{x\left[\mathrm{\mu g}/L\right]}{10}$
	Copper	Yttrium‐89	Helium gas	$4.75\times 10^{-2}\;\left[\mathrm{\mu g}/L\right]$	$y\left[\mathrm{\mu g}/\mathrm{dL}\right]=\frac{x\left[\mathrm{\mu g}/L\right]}{10}$

Abbreviations: ICP‐MS, inductively coupled plasma mass spectrometry; x, ICP‐MS measurement; y, standard method measurement.

^a^ Kanto Chemical (Tokyo, Japan).

### 2.5 Handling of Hemolyzed Samples

After centrifugation, serum samples, from which erythrocytes and other cellular components had been thoroughly removed, typically appear pale yellow color [48]. These serum samples were visually inspected and those with a reddish hue postcentrifugation were considered hemolyzed [49]. Among the 282 samples, hemolysis occurred in four samples prior to measurement using standard methods and in two samples that were first identified before ICP‐MS measurement. As the hemolysis in these cases was mild, these samples were generally included in the study. However, hemolysis can lead to an overestimation of certain analytes, as substances concentrated in erythrocytes and other cellular components may remain after centrifugation [50, 51]. To account for this, different approaches were adopted during the subsequent statistical analyses. Specifically, hemolyzed samples were actively included in analyses that tolerate the presence of outliers. Conversely, in analyses where outlier detection was considered, hemolyzed samples were initially excluded to statistically assess outliers due to factors other than hemolysis.

### 2.6 Descriptive Statistics and Statistical Analysis

When comparing the measurements obtained via standard methods to those obtained using ICP‐MS, the estimates of regression and correlation coefficients may become unstable if the variability of the explanatory variable is small [52]. Therefore, special attention was paid to the variability of each mineral in the descriptive statistics. In addition to the coefficient of variation, the quartile coefficient of dispersion (QCD) [53] was also employed as a representative measure of relative variability. The reason for specifically evaluating the latter is that most measurements in clinical laboratory analysis follow a non‐Gaussian distribution [54]. The assessment of deviation from a Gaussian distribution was based on the differences in skewness and kurtosis from zero. Moreover, the potential impact of differences in significant figures, specifically the rounding position of decimal values [55] on correlation, was considered. For non‐Gaussian distributions, central tendency and absolute variability were described using the range, key percentiles (5th and 95th), and quantile deviation. Gaussian measures such as the mean, standard deviation, and coefficient of variation are provided in S1.

Correlation analysis is commonly applied when comparing two variables. In this study, both Pearson's and Spearman's correlation coefficients were utilized. Pearson's correlation measures the linear association between variables, whereas Spearman's correlation is robust to non‐Gaussian distributions, allowing for the detection of relationships in data that deviate from normality [56]. If both coefficients exhibit similarly strong correlations, it suggests a stable relationship between variables. However, a stronger Spearman's correlation indicates potential deviations from a Gaussian distribution [56]. The correlation was categorized as very strong (≥ 0.9), strong (≥ 0.7), moderate (≥ 0.4), or weak (≥ 0.1) based on the lower limit associated with the 95% confidence interval (CI) of the Spearman correlation coefficients, which were interpreted using conventional methods [57].

While correlation coefficients are frequently used, they may not always yield appropriate conclusions in method comparison studies [58, 59]. Therefore, statistical and graphical techniques, such as Passing–Bablok regression, Deming regression, Mountain plots, and Bland–Altman plots, have been developed for such studies [59]. Among these, both Passing–Bablok regression and Bland–Altman plots are supported by the Validation‐Support/Excel software (version 61 and later), a freeware program based on Microsoft Excel (Microsoft Corporation, Redmond, Washington, USA) provided by the Quality Management Expert Committee of Japan Society of Clinical Chemistry for the validation of quantitative measurement methods [60]. These two methods are frequently used in conjunction in similar studies [61, 62]. In Japan, this freeware is occasionally employed for such purposes [63, 64]. Consistent with this practice, we opted to utilize both Passing–Bablok regression and Bland–Altman plots in our study. However, we did not use this freeware for our analysis owing to its limited graphical output capabilities.

Data from standard methods and ICP‐MS for serum mineral concentrations were compared to analyze concordance. Without excluding any outliers, including those from hemolyzed samples, Passing–Bablok regression yielded regression equations [59].

Bland–Altman plots were used to compare the mean and limits of agreement for the relative error, calculated as follows for each mineral [65]: $\frac{\mathrm{ICP}‐\mathrm{MS}\;\text{measurement}-\text{standard method measurement}}{\text{standard method measurement}}\times 100$.

The limits of agreement were the mean relative error ± 1.96 × standard deviation [66]. As relative error analysis assumes a Gaussian distribution and outliers can distort representative values, data filtered for outliers were used [67]. To identify outliers not caused by hemolysis, hemolyzed samples were first excluded, and then outliers were identified and filtered using standardized residuals greater than 3 from the Passing–Bablok regression, which is robust against outliers [59]. Additionally, a supplementary analysis was conducted to observe changes in the mean relative error and limits of agreement before and after filtering outliers.

A receiver operating characteristic (ROC) curve analysis was conducted to further evaluate the effectiveness of the ICP‐MS method in assessing mineral surpluses or deficiencies. Given the retrospective nature of this cross‐sectional study, the focus was on minerals with at least 10 cases of surplus or deficiency [68]. The area under the curve (AUC), sensitivity, and specificity were calculated, with cutoff values determined using Youden's index.

For descriptive statistics and statistical analyses, R software (version 4.33, R Foundation, Vienna, Austria) was used. As this study involves retrospective analysis of cross‐sectional data, instead of setting a significance level for hypothesis testing, 95% CIs for summary statistics and other statistical measures were calculated using the bootstrap method.

## 3 Results

### 3.1 Summary Statistics of Serum Minerals Quantitated Using Standard Methods

**FIGURE 1:**
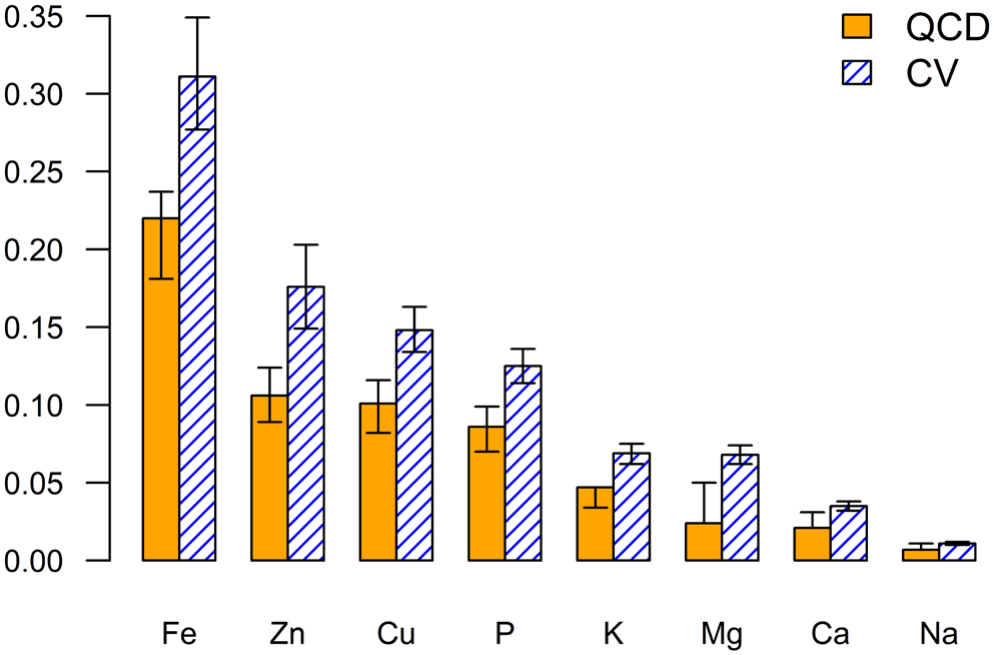
Comparison of the relative variability of the serum mineral dataset measured using standard methods. The bar plot shows the quartile coefficient of dispersion (QCD) and coefficient of variation (CV) for the eight serum minerals. Serum minerals are arranged in descending order of QCD. The error bars represent 95% confidence intervals. Ca, calcium; Cu, copper; Fe, iron; K, potassium; Mg, magnesium; Na, sodium; P, phosphorus; Zn, zinc.

Tables 3 and S3 present the summary statistics for serum mineral levels measured using standard methods. These minerals exhibited varying degrees of central tendencies and variability. Figure 1 compares the relative variability of each serum mineral. Notably, sodium, calcium, and magnesium, which demonstrated the smallest relative variability, possessed lower kurtosis values, indicating shorter distribution tails on both sides. Conversely, iron and zinc, which had higher relative variabilities, exhibited higher positive skewness, suggesting a longer right tail in their distributions.

**TABLE 3:** Summary statistics for serum mineral screening dataset measured using standard methods.

Serum mineral	Min	5%ile	25%ile	50%ile	75%ile	95%ile	Max	QD	QCD (95% CI)	Skewness	Kurtosis	RIs	Below LLR [%]	Above ULR [%]
Na [mmol/L]^a^	137	139	140	141	142	144	146	1.0	0.00709 (0.00709, 0.0106)	0.17	0.05	138–145	0.4	0.7
K [mmol/L]^b^	3.3	3.9	4.1	4.3	4.5	4.9	5.3	0.20	0.0465 (0.0345, 0.0465)	0.33	0.91	3.6–4.8	0.7	6.4
Ca [mg/dL]^b^	8.8	9.1	9.4	9.6	9.8	10.2	10.7	0.20	0.0208 (0.0208, 0.0312)	0.34	−0.04	8.8–10.1	0.0	5.3
P [mg/dL]^b^	2.0	2.7	3.2	3.5	3.8	4.1	4.7	0.30	0.0857 (0.0704, 0.0986)	−0.15	0.19	2.7–4.6	2.8	0.4
Mg [mg/dL]^b^	1.7	1.8	2.0	2.1	2.1	2.3	2.4	0.050	0.0244 (0.0244, 0.0476)	0.07	0.00	1.8–2.5	1.8	0.0
Fe [μg/dL]^a^	29	59	77	100	121	159	278	21.8	0.220 (0.183, 0.239)	0.90	2.75	40–188	0.7	0.7
Zn [μg/dL]^a^	50	62	72	79	89	100	161	8.5	0.106 (0.0886, 0.121)	1.67	6.74	80–130	52.1	1.8
Cu [μg/dL]^a^	58	72	83	92	102	117	153	9.4	0.101 (0.0820, 0.114)	0.59	0.89	71–132	2.8	0.4

Abbreviations: Ca, calcium; CI, confidence interval; Cu, copper; Fe, iron; K, potassium; LLR, lower limit of reference intervals; Max, maximum; Mg, magnesium; Min, minimum; Na, sodium; P, phosphorus; QCD, quartile coefficient of dispersion, which describes relative variability; QD, quantile deviation, which describes absolute variability; RIs, Reference intervals; ULR, upper limit of reference intervals; Zn, zinc; %ile, percentile, which describes central tendency or absolute variability.

^a^ First decimal is rounded as precision is maintained to the integer part for measurements.

^b^ Second decimals are rounded as precision is maintained to the first decimal place for measurements.

Table 3 depicts the proportion of participants whose serum mineral levels were either deficient (below the lower limit of reference [LLR] intervals) or surplus (above the upper limit of reference [ULR] intervals). The number of participants with deficiencies or surpluses exceeding 10 was notably high for those with serum zinc deficiency (135 participants, 52.1%), followed by participants with surplus serum potassium (18 participants, 6.40%), and surplus serum calcium (15 participants, 5.30%). The proportions of deficiencies or surpluses for other serum minerals were below 10 participants (3.55%).

### 3.2 Comparison of Standard and ICP‐MS Methods

**FIGURE 2:**
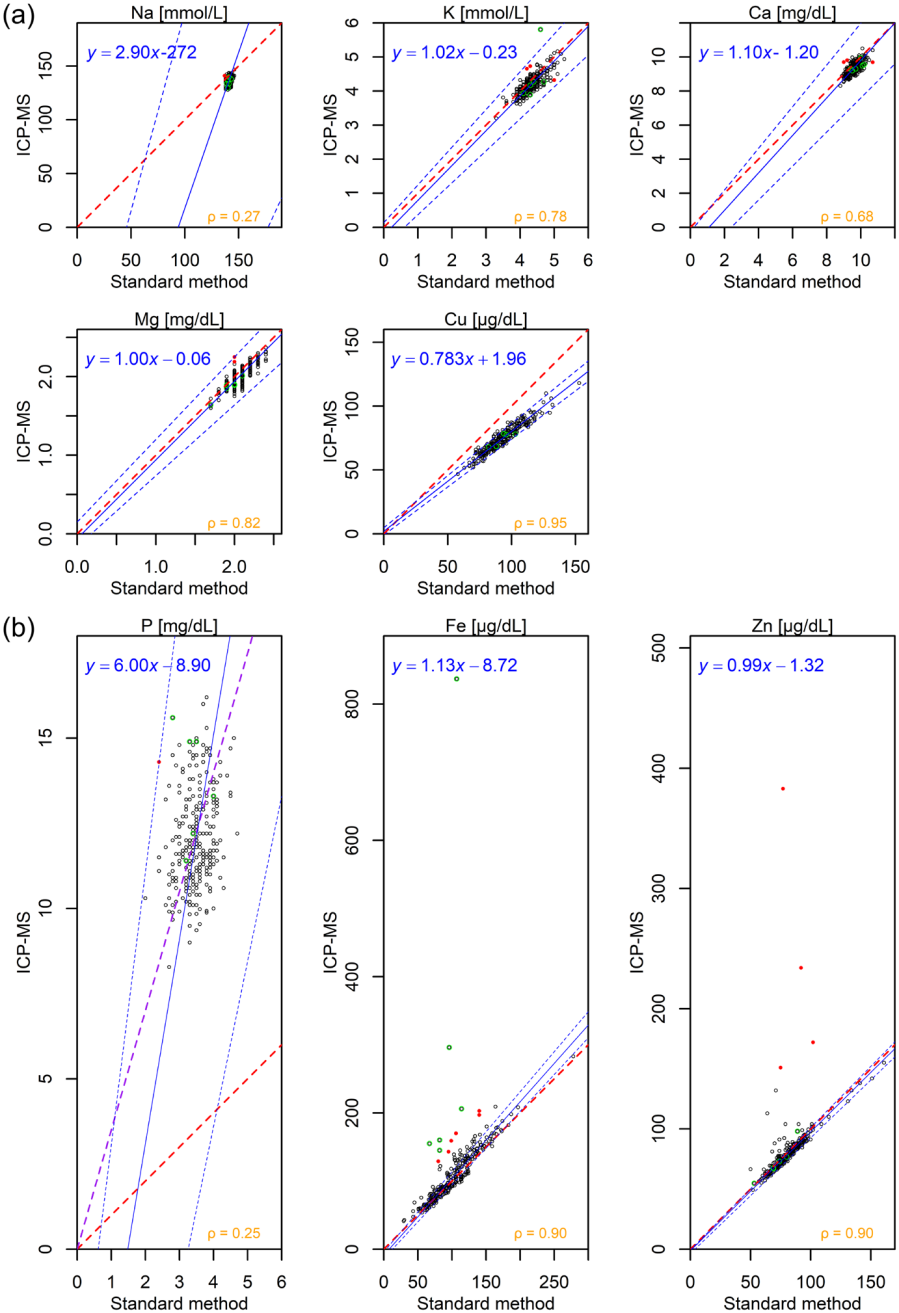
Scatter plots and Passing–Bablok regression lines comparing serum mineral concentrations measured using the ICP‐MS and standard methods. The eight scatter plots represent the measurements for all data (*n* = 282), with the vertical axis showing the ICP‐MS measurements and the horizontal axis showing the standard‐method measurements on a consistent scale. Owing to the lower number of significant digits in the standard method measurements than those for ICP‐MS, the plots exhibit a discrete pattern along the horizontal axis. Sodium (Na), potassium (K), calcium (Ca), magnesium (Mg), and copper (Cu), which exhibited minimal scale differences between the ICP‐MS and standard methods, are plotted with a 1:1 ratio between the vertical and horizontal axes (a). Phosphorus (P), iron (Fe), and zinc (Zn), which exhibited higher variance in ICP‐MS than in the standard method, are plotted with an aspect ratio of 3:1 (b), without changing the scale of the axes. The solid blue line represents the Passing–Bablok regression estimated from all the data, whereas the dashed blue lines indicate the upper and lower 95% confidence intervals. Green‐circled points represent hemolyzed samples. Red points indicate outliers due to factors other than hemolysis. These outliers have standardized residuals > 3 when comparing the theoretical ICP‐MS measurements, derived from the Passing–Bablok regression, with the actual ICP‐MS measurements, excluding hemolyzed samples. The regression equations are displayed in the top‐left corner of each plot. The dashed red line represents the identity line, where the slope is 1.00 and the intercept is 0.00. For P, the dashed purple represents a slope of 3.50 and an intercept of 0.00, supplementing the identity line. ICP‐MS, inductively coupled plasma mass spectrometry.

Figure 2 and Table S4 compare the standard method and ICP‐MS using Passing–Bablok regression. The regression lines, derived without excluding outliers, are indicated as robust lines. Notably, Figure 2 was adjusted to include zero on the *x*‐axis, representing measurements obtained using the standard method, to clearly illustrate the interindividual variability across minerals. Additionally, the *y*‐axis scale was not truncated to depict the number and extent of outliers measured using ICP‐MS. A zoomed‐in version of the plot around the regression line is provided in Figure S2. Agreement between methods is indicated if the 95% CI for the intercept includes 0 and the slope includes 1; magnesium and zinc met these criteria. Potassium, calcium, and iron had slopes close to 1 but slightly negative intercepts. For copper, its intercept included 0; however, its slope (95% CI: 0.753–0.817) indicated that ICP‐MS measurements were 25%–30% lower. For sodium, despite regression instability, the median value was 141 mmol/L (IQR: 140–142 mmol/L) and 137.9 mmol/L (IQR: 135.7–140.0 mmol/L) when measured using the standard method and ICP‐MS, respectively, suggesting minimal scale bias despite errors. Comparing the 95% CI of Spearman's ρ values, copper exhibited a very strong correlation; potassium, magnesium, iron, and zinc exhibited a strong correlation; calcium exhibited a moderate correlation; and sodium demonstrated a weak correlation (Table S4). Although Pearson's *r* and Spearman's *ρ* values were similar for most minerals, outliers led to a reduction in Pearson's *r* values for iron and zinc (Table S4).

Figures 2 and S3 highlight outliers for each mineral. All six hemolyzed samples exhibited significant overestimation in iron, and one sample also displayed a similar issue with potassium. For the other elements, most hemolyzed samples clustered with the main group, indicating minimal impact of hemolysis. Nonhemolytic outliers, presented as red points in the figure, were more common in iron and zinc (six and four samples, respectively) as ICP‐MS overestimations, whereas underestimations were less common (one each in potassium and calcium) (Figures 2 and S3).

For phosphorus, the standard method exhibited a median of 3.5 mg/dL (IQR: 3.2–3.8 mg/dL), whereas ICP‐MS had a median of 11.8 mg/dL (IQR: 11.0–13.0 mg/dL), indicating a proportional bias reflecting differences between serum inorganic and total phosphorus concentrations (slope 95% CI: 4.85–8.00) (Figure 2 and Table S4). Ignoring the intercept, ICP‐MS values were approximately 3.5 times higher than those obtained using standard methods. The weak correlation between standard and ICP‐MS phosphorus measurements suggests a relationship between inorganic and total phosphorus, indicating that although the regression equation is unstable, it remains partially informative (Table S4).

### 3.3 Comparison of Relative Errors Between Standard and ICP‐MS Methods for Each Mineral

**FIGURE 3:**
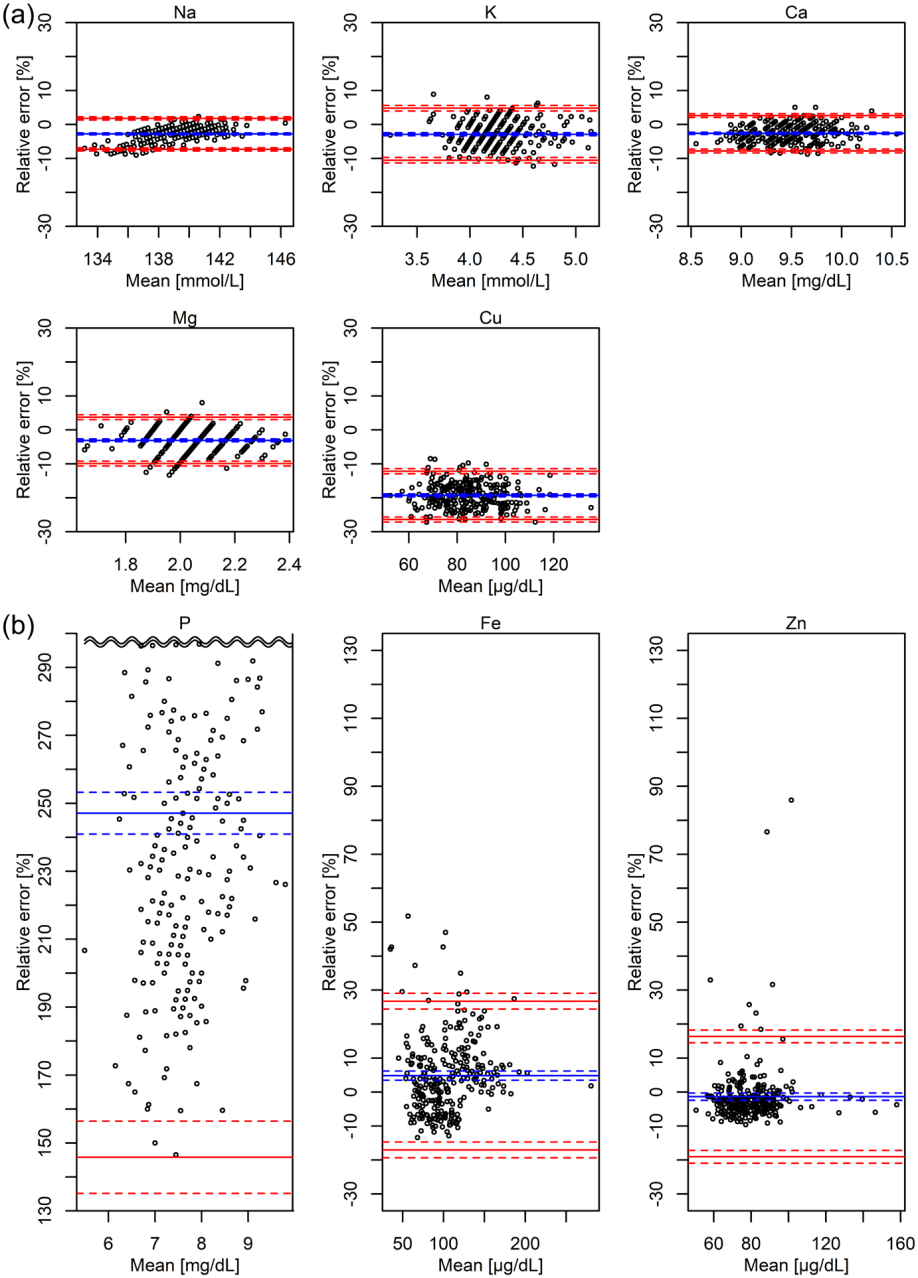
Bland–Altman plots of the relative errors between ICP‐MS and standard methods. The eight scatter plots display data that were filtered using Passing–Bablok regression analysis, first excluding six hemolyzed samples, followed by filtering outliers due to non‐hemolysis factors. The vertical axis represents the relative error [%], calculated as $\frac{\mathrm{ICP}‐\mathrm{MS}\;\text{measurement}-\text{standard method measurement}}{\text{standard method measurement}}\times 100$, and the horizontal axis represents the mean, calculated as $\frac{\mathrm{ICP}‐\mathrm{MS}\;\text{measurement}+\text{standard method measurement}}{2}$. The number of outliers due to nonhemolysis factors excluded for each mineral—sodium, potassium, calcium, phosphorus, magnesium, iron, zinc, and copper—are 1, 3, 3, 1, 2, 6, 4, and 0, respectively. The vertical axis scale is consistent across all minerals; however, for sodium, potassium, calcium, magnesium, and copper in (a) the relative error is within 30%, whereas for phosphorus, iron, and zinc in (b) the relative error exceeds 30%, necessitating a wider display range. The solid blue and dashed lines represent the mean relative errors and 95% confidence intervals, respectively. The solid red and dashed lines represent the lower and upper limits of agreement and 95% confidence intervals, respectively. For phosphorus, owing to space constraints, only the lower half of the mean relative error is shown, with part of the upper half omitted, but it is approximately symmetrical. The lower and upper limits of agreement were calculated as the mean relative error ± 1.96 × the standard deviation of the relative error. ICP‐MS, inductively coupled plasma mass spectrometry; *ρ*, Spearman's correlation coefficient.

The relative errors between the standard and ICP‐MS methods were subsequently compared for each mineral. Figure 3 presents the relative errors and limits of the agreement after filtering hemolysis samples and outliers via Bland–Altman plots. The changes before and after filtering are detailed in Table S5. Sodium, potassium, calcium, and magnesium exhibited mean relative errors of approximately −3% with limits of agreement ranging from −12% to +6% (Figure 3 and Table S5). This result indicates that ICP‐MS measurements tended to be slightly lower than those obtained using standard methods. Copper demonstrated a mean relative error of −19% with limits of agreement ranging from −26% to −12% (Figure 3 and Table S5), indicating a downward bias in ICP‐MS measurements. Phosphorus had a mean relative error of approximately 250% (Figure 3 and Table S5), reflecting the difference between total phosphorus measured using ICP‐MS and inorganic phosphorus measured using the standard method.

Despite the systematic exclusion of outliers, several outliers exceeding the upper limit of agreement persisted for iron and zinc (Figure 3). After filtering, the mean relative errors for iron and zinc were approximately +5% and 0%, respectively, with limits of agreement around −20% to +30% for iron and − 20% to +15% for zinc (Figure 3 and Table S5). This result suggests that ICP‐MS occasionally overestimates these minerals. The significant differences in iron and zinc were due to large outliers with standardized residuals greater than 5, with iron primarily affected by hemolysis and zinc by nonhemolysis factors (Figure S3). In contrast, filtering caused little change in the mean relative errors and limits of agreement for sodium, potassium, calcium, phosphorus, magnesium, and copper (Table S5), indicating that their relative errors generally followed a Gaussian distribution.

### 3.4 Evaluation of ICP‐MS Effectiveness in Detecting Mineral Surplus or Deficiency

**FIGURE 4:**
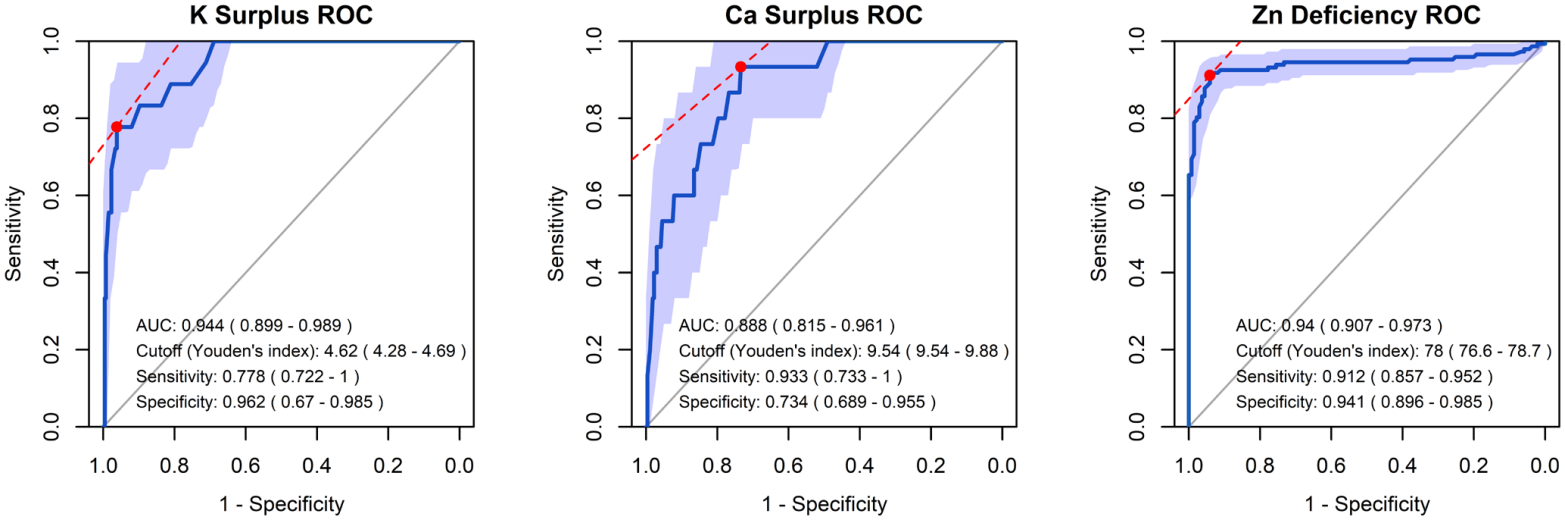
ROC curves for predicting three different conditions. The ROC curves were plotted to predict three different conditions: potassium (K) surplus, calcium (Ca) surplus, and zinc (Zn) deficiency. The ROC curves were generated based on all data (*n* = 282), including outliers caused by hemolysis or other factors. The potassium surplus group, calcium surplus, and zinc deficiency groups included 18, 15, and 135 individuals, respectively. The light blue shaded area represents the 95% confidence interval of the ROC curve. The red dot indicates the point with the minimum distance to the top‐left corner, determined using Youden's index. The numbers in parentheses represent the 95% confidence intervals for the AUC, cutoff, sensitivity, and specificity. AUC, area under the curve; ICP‐MS, inductively coupled plasma mass spectrometry; ROC, receiver operating characteristic.

Finally, the detection capability of ICP‐MS for mineral surpluses and deficiencies was evaluated. ROC analysis was conducted for potassium surplus, calcium surplus, and zinc deficiency, each having more than 10 cases. Although the sample sizes for potassium and calcium surpluses were smaller than those for zinc deficiency, leading to slightly unstable 95% CIs for the AUC, all three exhibited AUC values of approximately 0.9 (Figure 4). This result indicates that ICP‐MS demonstrates adequate performance for screening these conditions.

## 4 Discussion

In this study, the serum levels of eight key minerals commonly measured in Japan were compared using ICP‐MS and standard methods to reveal the specific characteristics of the ICP‐MS method. First, stable regression coefficients were observed for most minerals except phosphorus, which was measured differently, and sodium, where the regression analysis was unstable. Second, a weak correlation was noted between the serum total phosphorus concentration measured using ICP‐MS and the serum inorganic phosphorus concentration measured using the standard method. Third, extreme outliers were observed for iron and zinc, which tended to lead to significant overestimation in the regression line. The outliers for iron were primarily due to hemolysis, whereas those for zinc were likely attributed to factors other than hemolysis.

The present results indicated that except for phosphorus, minerals with larger relative variability tended to yield more stable regression equations between ICP‐MS and standard methods. Specifically, minerals such as iron, zinc, and copper, which exhibited relatively large interindividual variability in serum concentrations (Figure 1), demonstrated narrow CIs for the regression coefficients and strong correlation coefficients (Figure 2). Conversely, sodium, which demonstrated significant instability in regression analysis, may exhibit errors attributable to calibration issues or matrix effects from other components in the samples [69]. However, the primary factor likely contributing to the poor agreement is the narrow optimal range and minimal interindividual variability in serum sodium levels, which are strictly regulated. Despite the smaller relative variability and weaker correlation coefficients for sodium, the mean relative error was approximately −3% (Figure 3), comparable to that of potassium and magnesium, which had higher correlation coefficients. Notably, even calcium, which has the next smallest relative variability after sodium, proved useful for detecting calcium surplus (Figure 4). These findings suggest that while the relative errors for abundant serum minerals such as sodium, potassium, calcium, and magnesium are minimized by ICP‐MS, caution is required when interpreting ICP‐MS measurements for minerals with low interindividual variability.

Among the minerals examined, copper yielded the most robust regression equation. Notably, this result is consistent with previous reports [70, 71]. However, it was also the mineral for which the slope of the regression line was significantly less than 1 (Figure 2). Although the measurement of serum copper using ICP‐MS exhibits excellent correlation and agreement with conventional methods [72], the matrix effects can be pronounced, leading to potential negative bias, especially when using internal standard methods [73]. Additionally, signal suppression due to the space charge effect may also account for this discrepancy [74].

In the present study, the total serum phosphorus concentration measured using ICP‐MS was approximately 3.5 times higher than that determined based on the inorganic phosphate concentration measured using the standard method (Figure 2). This result aligns with the fact that inorganic phosphate accounts for only 30% of circulating phosphorus, while the remaining 70% consists primarily of organic phosphorus compounds [28], such as lipid phosphorus and phosphoric esters [75]. Organic phosphorus compounds are essential for key cellular processes, such as energy metabolism, redox state, and cell signaling [76], underscoring the importance of focusing on phosphorus fractions rather than on the absolute value of total phosphorus [77]. Historically, total phosphorus was commonly measured until the 1960s to allow the simultaneous evaluation of nucleic acids [78] and phospholipids [79]. Even in the 21st century, there have been attempts to consider total phosphorus concentration with an emphasis on its different fractions [80]. However, only a few studies have examined the relationship between total phosphorus and inorganic phosphorus in human serum [77, 81]. In this context, our study provides valuable data demonstrating the correlation between serum total phosphorus levels and serum inorganic phosphorus levels in humans. Additionally, compared with our findings, those from previous studies that measured total phosphorus concentrations in different populations, revealed higher [77], equivalent [82], and lower [13, 83, 84, 85] values. This discrepancy is likely attributed to differences in race, region, diet, or analytical methods.

In the present study, hemolysis effects were evident in the ICP‐MS measurements of iron and some potassium (Figure 2). Hemolysis, which involves the release of intracellular fluid from erythrocytes into plasma, can lead to an overestimation of potassium, phosphorus, magnesium, iron, and zinc levels [50, 51]. Therefore, in samples with significant erythrocyte destruction, these minerals should produce outliers simultaneously, regardless of the measurement method, as substances normally removed during centrifugation are instead released into the serum. Notably, in our study, the effects of hemolysis differed between the standard method and ICP‐MS, predominantly skewed toward iron measurements. This discrepancy might be attributed to differences in the measurement targets of the two methods, particularly for iron. While the standard methods typically measure only transferrin‐bound iron [86], a preprint available on PubMed indicates that ICP‐MS measures the total iron content, including both transferrin‐bound and free hemoglobin iron [87]. This may explain the significant discrepancy observed in iron measurements. Our literature search did not reveal similar findings for sodium, potassium, calcium, magnesium, or zinc. Excluding phosphorus, where the measurement targets are known to differ, this suggests that iron may be the only mineral measured in this study to exhibit such differences between ICP‐MS and the standard method. Given that approximately two‐thirds of transferrin remains unbound to iron [88], in cases of significant hemolysis where erythrocytes and hemoglobin are disrupted, the iron released from erythrocytes would eventually bind to transferrin and thus be detectable using standard methods. However, in cases of very mild hemolysis, where the iron remains bound within free hemoglobin, this iron may not bind to transferrin and thus remain undetected using standard methods. In contrast, ICP‐MS may exhibit greater sensitivity to such minor increases in free hemoglobin iron. Furthermore, even after excluding visibly hemolyzed samples, some ICP‐MS iron measurements were slightly overestimated compared with those obtained using standard methods (Figures 2 and 3). This discrepancy may reflect subtle increases in free hemoglobin that are visually undetectable and likely originate from erythrocytes.

The occurrence of outliers in zinc measurements due to nonhemolysis factors is not well understood (Figure 2). Although standard methods for zinc measurement have been criticized for inaccuracy [72, 89], and ICP‐MS is generally regarded as more reliable, our study noted some extreme outliers. Typically, phenomena leading to abnormally high ICP‐MS results include the addition of polyatomic ions that helium gas cannot fully remove [16], the presence of doubly charged ions such as barium ions [16], and carry‐over effects due to inadequate cleaning of the analytical pathways [69]. However, these represent random errors and may not sufficiently explain the outliers that deviate significantly from a Gaussian distribution. Through a process of elimination, one possible cause for this result is contamination during sample handling. In this study, blood samples were collected and promptly measured by clinical laboratory technologists following strict protocols. However, for ICP‐MS, the samples were handled and frozen by urologists during clinical duties, which might have introduced exogenous contamination. Notably, zinc is ubiquitous in laboratories, and contamination from sources such as single‐use gloves has been reported [90].

The present study analyzed cross‐sectional data from a real‐world setting, revealing that ICP‐MS exhibited generally low measurement errors (Figure 3) and reasonable diagnostic performance for detecting surplus or deficiencies (Figure 4). However, it also revealed that precision could decline for minerals with small relative variations, such as sodium, and that unexpected outliers could arise for elements such as iron owing to the inherent characteristics of ICP‐MS (Figure 2). These findings highlight the need for further validation before ICP‐MS can be fully utilized as a complete alternative to standard methods for single minerals. The advantage of ICP‐MS lies in its ability to simultaneously measure trace elements across a wide range of scales, rather than being limited to a single mineral [91, 92, 93, 94, 95]. To demonstrate the application of this advantage, the effectiveness of machine learning approaches that consider the complex interrelationships among multiple parameters has been reported [10, 96]. We have previously applied machine learning techniques to this cross‐sectional dataset and proposed a novel classification method for male infertility [21]. By adopting a multivariate simultaneous analysis approach, mitigating the potential limitations of ICP‐MS identified in this study, such as instability in the precision of single mineral measurements and the occasional occurrence of outliers for individual minerals, may be feasible, further emphasizing the importance of such approaches.

Another notable aspect of this study is that ICP‐MS was used to simultaneously measure both relatively high‐concentration and trace minerals. Traditional ICP‐MS systems recommend limiting total dissolved solids (TDS) content to below 0.2% [16], which is considerably lower than the 0.9% sodium chloride concentration (including 154 mmol/L sodium ions) in normal saline [97]. To accommodate high‐TDS samples, dual instrumentation involving both ICP‐OES and ICP‐MS is often employed [98, 99]. However, recent advancements in ICP‐MS technology, including the instrument used in this study, support up to 3% TDS [100], with newer models handling up to 25% [101], thus approaching the 30% support of the latest ICP‐OES models [102]. This could eventually minimize concerns over high‐TDS content in ICP‐MS. Although ICP‐OES is generally considered a more economical option for elements that do not require the lower detection limits offered by ICP‐MS [103, 104], we believe that using ICP‐MS alone for comprehensive multimineral measurements optimizes operational efficiency and provides better overall cost‐effectiveness when compared to dual instrumentation. Additionally, ICP‐OES cannot detect low‐concentration minerals with the same sensitivity as ICP‐MS [16], further highlighting the importance of using ICP‐MS for both high‐ and low‐concentration mineral detection. Our laboratory continues to optimize these systems to improve the simultaneous measurement of high‐ and low‐concentration minerals, and future evaluations will focus on the cost‐effectiveness of this approach.

This study had several limitations. First, the study population was limited to patients visiting a specific hospital in Japan, which may limit the generalizability of the findings. Second, owing to the generally good health status of the study participants and the retrospective nature of the cross‐sectional analysis, the evaluation of diagnostic performance for conditions other than zinc deficiency, which is notably more common among the Japanese [105], was restricted to a very small number of cases involving potassium excess and calcium excess. Third, the study design involved initial sample handling by urologists who were not specialized in chemical analysis, potentially preventing the full realization of the capabilities of ICP‐MS compared with other studies. Fourth, trace minerals that are not commonly measured in routine clinical practice were not examined. While ICP‐MS is attractive for its ability to comprehensively measure multiple types of minerals simultaneously, the cross‐sectional study design limited the investigation to eight minerals. Therefore, future research should include larger sample sizes and cohorts with diverse mineral‐related profiles to provide more comprehensive validation.

## 5 Conclusion

This study provides a strong foundation for future research by evaluating the performance of ICP‐MS using real‐world data. The study provides insights that not only clarify the characteristics of serum mineral measurements commonly encountered in routine clinical practice but also highlight the potential of ICP‐MS to advance comprehensive multiparametric analysis and pioneer new approaches in clinical diagnostics.

## Author Contributions

Kosuke Kojo was primarily responsible for conceptualization, data curation, formal analysis, funding acquisition, investigation, methodology, visualization, and writing of the original draft. Tomoko Oguri was responsible for data curation, methodology, validation, and writing of review and editing. Takazo Tanaka was primarily responsible for formal analysis and investigation. Atsushi Ikeda was primarily responsible for conceptualization. Takuya Shimizu and Shunsuke Fujimoto were primarily responsible for resources and software. Ayumi Nakazono was primarily responsible for the investigation. Yoshiyuki Nagumo, Shuya Kandori, Hiromitsu Negoro, and Hiroyuki Nishiyama were primarily responsible for supervision. All authors were involved in the interpretation of data for the work, critical revision of the work for important intellectual content, final approval of the version to be published, and agreement to be accountable for all aspects of the work to ensure that questions related to the accuracy or integrity of any part of the work are appropriately investigated and resolved.

## Ethics Statement

All procedures were performed in accordance with the 1964 Declaration of Helsinki and its later amendments and were approved by the Ethical Committee of Tsukuba University Hospital (approval numbers: #H30‐152 and #R03‐127).

## Consent

Informed consent was obtained from all participants.

## Conflicts of Interest

The authors declare no conflicts of interest.

## Supporting information


Data S1.

## Data Availability

The data underlying this article will be shared upon reasonable request from the corresponding author.
