# Supplementary material for: Inductively Coupled Plasma Mass Spectrometry Performance for the Measurement of Key Serum Minerals: A Comparative Study With Standard Quantification Methods
Source: J Clin Lab Anal. 2024 Dec 23;39(2):e25140. doi: 10.1002/jcla.25140 (PMC11776499; doi:10.1002/jcla.25140)

Inductively Coupled Plasma Mass Spectrometry Performance for the Measurement of Key Serum Minerals: A Comparative Study with Standard Quantification Methods

Supplementary Information

**Page 2.**	**Supplementary Table S1.**	Age distribution of participants.
**Page 3.**	**Supplementary Table S2.**	Certified standard reference ranges for Seronorm Trace Elements Serum Level 1 and measured values obtained using our ICP-MS measurement method.
**Page 4.**	**Supplementary Table S3.**	Summary statistics for the serum mineral screening dataset measured using standard methods.
**Page 5.**	**Supplementary Table S4.**	Comparison of measurements made using the ICP-MS and standard methods with Passing–Bablok regressions, and their correlation coefficients.
**Page 6.**	**Supplementary Table S5.**	Comparison of the mean relative errors and limits of agreement between ICP-MS and standard methods for all data, after excluding hemolysis samples and filtering of outliers.
**Page 7.**	**Supplementary Figure S1.**	Classification of the fertility of participants.
**Page 8.**	**Supplementary Figure S2.**	Scatter plots and Passing–Bablok regression lines comparing serum mineral concentrations measured using the ICP-MS and standard methods.
**Page 9.**	**Supplementary Figure S3.**	Scatter plots of standardized residuals between theoretical ICP-MS measurements estimated from Passing–Bablok regression and actual ICP-MS measurements.

Supplementary Table S1. Age distribution of participants.

Age range (years)	Number of participants (%)
20–24	5 (1.8)
25–29	39 (13.8)
30–34	80 (28.4)
35–39	73 (25.9)
40–44	48 (17.0)
45–49	29 (10.3)
50–54	5 (1.8)
55–59	1 (0.4)
60–64	1 (0.4)
65–70	0 (0.0)
75–79	1 (0.4)

Supplementary Table S2. Certified standard reference ranges for Seronorm Trace Elements Serum Level 1 and measured values obtained using our ICP-MS measurement method.

Serum mineral	Certified range	Measured value	
		Mean (SD)	CV (number)
**Na [mg/L]**	2330–3504	2842 (45.8)	0.016 (10)
**K [mg/L]**	101–153	125 (2.32)	0.019 (10)
**Ca [mg/L]**	69–104	86.5 (1.57)	0.018 (10)
**P [mg/L]**	43.3–65.1	45.0 (1.00)	0.022 (10)
**Mg [mg/L]**	13.4–20.1	16.2 (0.49)	0.030 (10)
**Fe [μg/L]**	1170–1770	1419 (72.7)	0.051 (10)
**Zn [μg/L]**	952–1242	1078 (13.6)	0.013 (10)
**Cu [μg/L]**	999–1176	1045 (15.3)	0.015 (10)

ICP-MS, inductively coupled plasma mass spectrometry; SD, standard deviation; CV, coefficient of variation; Na, sodium; K, potassium; Ca, calcium; P, phosphorus; Mg, magnesium; Fe, iron; Zn, zinc; Cu, copper.

Supplementary Table S3. Summary statistics for the serum mineral screening dataset measured using standard methods.

Serum mineral	$\bar{x}$-3σ	$\bar{x}$-2σ	$\bar{x}$-1σ	$\bar{x}$	$\bar{x}$+1σ	$\bar{x}$+2σ	$\bar{x}$+3σ	σ	CV (95%CI)
**Na [mmol/L] †**	136.4	138.0	139.6	141.2	142.8	144.4	146.0	1.61	0.0114 (0.0105, 0.0124)
**K [mmol/L] ‡**	3.44	3.74	4.03	4.33	4.63	4.92	5.22	0.296	0.0685 (0.0613, 0.0748)
**Ca [mg/dL] ‡**	8.59	8.93	9.26	9.60	9.94	10.28	10.62	0.338	0.0353 (0.0323, 0.0380)
**P [mg/dL] ‡**	2.18	2.61	3.05	3.49	3.92	4.36	4.80	0.437	0.125 (0.114, 0.136)
**Mg [mg/dL] ‡**	1.64	1.78	1.92	2.06	2.20	2.34	2.48	0.140	0.0679 (0.0624, 0.0738)
**Fe [μg/dL] †**	7.1	38.8	70.6	102.4	134.1	165.9	197.6	31.8	0.311 (0.277, 0.350)
**Zn [μg/dL] †**	38.1	52.3	66.4	80.5	94.7	108.8	122.9	14.1	0.176 (0.147, 0.201)
**Cu [μg/dL] †**	51.7	65.5	79.2	93.0	106.7	120.5	134.2	13.8	0.148 (0.133, 0162)

$\bar{x}$, mean, which describes the central tendency; σ, standard deviation, which describes the absolute variability; CV, coefficient of variation, which describes the relative variability; CI, confidence interval; Na, sodium; K, potassium; Ca, calcium; P, phosphorus; Mg, magnesium; Fe, iron; Zn, zinc; Cu, copper.

† The first decimal place is described for reference although precision was maintained to the integer part for measurements

‡ The second decimal place is described for reference, although precision is maintained to the first decimal place for measurements

Supplementary Table S4. Comparison of measurements made using the ICP-MS and standard methods with Passing–Bablok regressions, and their correlation coefficients.

**Serum mineral**	**Passing–Bablok regressions**		**Correlation coefficients**	
	**Intercept (95%CI)**	**Slope (95%CI)**	**Pearson's *r* (95%CI)**	**Spearman's *ρ* (95%CI)**
**Na [mmol/L]**	−272.2 (−385.0, −169.6)	2.90 (2.17, 3.70)	0.268 (0.156, 0.373)	0.270 (0.158, 0.375)
**K [mmol/L]**	−0.230 (−0.597, −0.136)	1.023 (0.938, 1.108)	0.785 (0.735, 0.826)	0.781 (0.731, 0.823)
**Ca [mg/dL]**	−1.20 (−2.41, −0.26)	1.10 (1.00, 1.23)	0.698 (0.633, 0.754)	0.683 (0.615, 0.741)
**P [mg/dL]**	−8.90 (−15.80, −4.93)	6.00 (4.85, 8.00)	0.245 (0.132, 0.352)	0.247 (0.134, 0.354)
**Mg [mg/dL]**	−0.06 (−0.16, 0.15)	1.00 (0.90, 1.05)	0.849 (0.812, 0.878)	0.818 (0.775, 0.853)
**Fe [µg/dL]**	−8.721 (−13.70, −3.960)	1.125 (1.080, 1.175)	0.580 (0.497, 0.653)	0.903 (0.879, 0.923)
**Zn [µg/dL]**	−1.322 (−3.638, 0.738)	0.989 (0.963, 1.019)	0.517 (0.426, 0.598)	0.896 (0.871, 0.917)
**Cu [µg/dL]**	1.958 (−0.975, 4.668)	0.783 (0.753, 0.817)	0.953 (0.941, 0.963)	0.948 (0.935, 0.959)

ICP-MS, inductively coupled plasma mass spectrometry; CI, confidence interval; Na, sodium; K, potassium; Ca, calcium; P, phosphorus; Mg, magnesium; Fe, iron; Zn, zinc; Cu, copper.

Supplementary Table S5. Comparison of the mean relative errors and limits of agreement between ICP-MS and standard methods for all data, after excluding hemolysis samples and filtering of outliers.

Serum mineral	Outlier inclusion	Number	LLoA‡ (95%CI) [%]	Mean RE† (95%CI) [%]	ULoA‡ (95%CI) [%]
**Na**	All	282	−7.40 (−7.88, −6.92)	−2.79 (−3.06, −2.51)	1.83 (1.35, 2.31)
	Excl. hemolysis	276	−7.37 (−7.86, −6.89)	−2.76 (−3.04, −2.48)	1.85 (1.37, 2.34)
	Filtered	275	−7.35 (−7.83, −6.87)	−2.78 (−3.06, −2.50)	1.79 (1.31, 2.27)
**K**	All	282	−11.5 (−12.4, −10.6)	−2.77 (−3.29, −2.25)	5.95 (5.05, 6.85)
	Excl. hemolysis	276	−10.9 (−11.7, −10.0)	−2.82 (−3.31, −2.33)	5.22 (4.38, 6.06)
	Filtered	273	−10.5 (−11.4, −9.74)	−2.88 (−3.34, −2.41)	4.79 (3.98, 5.60)
**Ca**	All	282	−8.06 (−8.63, −7.49)	−2.58 (−2.91, −2.25)	2.91 (2.34, 3.47)
	Excl. hemolysis	276	−8.06 (−8.63, −7.48)	−2.56 (−2.89, −2.22)	2.94 (2.37, 3.52)
	Filtered	273	−7.82 (−8.37, −7.27)	−2.60 (−2.92, −2.29)	2.61 (2.06, 3.16)
**P**	All	282	141 (130, 153)	249 (243, 256)	357 (346, 369)
	Excl. hemolysis	276	143 (132, 154)	248 (242, 254)	353 (342, 364)
	Filtered	275	146 (135, 156)	247 (241, 253)	348 (338, 359)
**Mg**	All	282	−10.1 (−10.9, −9.39)	−2.99 (−3.42, −2.57)	4.14 (3.40, 4.88)
	Excl. hemolysis	276	−10.2 (−10.9, −9.42)	−2.98 (−3.42, −2.55)	4.21 (3.46, 4.97)
	Filtered	274	−9.90 (−10.6, −9.19)	−3.08 (−3.50, −2.67)	3.74 (3.02, 4.45)
**Fe**	All	282	−78.4 (−87.6, −69.2)	10.3 (4.98, 15.6)	98.9 (89.8, 108)
	Excl. hemolysis	276	−19.9 (−22.6, −17.2)	5.88 (4.32, 7.44)	31.7 (29.0, 34.4)
	Filtered	270	−17.1 (−19.4, −14.7)	4.83 (3.50, 6.17)	26.7 (24.4, 29.0)
**Zn**	All	282	−53.5 (−59.1, −47.8)	1.26 (−2.01, 4.53)	56.0 (50.3, 61.7)
	Excl. hemolysis	276	−54.0 (−59.8, −48.2)	1.28 (−2.07, 4.62)	56.6 (50.8, 62.4)
	Filtered	272	−19.1 (−20.9, −17.2)	−1.36 (−2.44, −0.278)	16.4 (14.5, 18.2)
**Cu**	All	282	−26.4 (−27.1, −25.7)	−19.3 (−19.7, −18.9)	−12.2 (−13.0, −11.5)
	Excl. hemolysis	276	−26.4 (−27.2, −25.7)	−19.3 (−19.7, −18.9)	−12.2 (−12.9, −11.4)
	Filtered	276	−26.4 (−27.2, −25.7)	−19.3 (−19.7, −18.9)	−12.2 (−12.9, −11.4)

ICP-MS, inductively coupled plasma mass spectrometry; LLoA, lower limit of agreement; CI, confidence interval; RE, relative error; ULoA, upper limit of agreement; Na, sodium; Excl., after excluding; K, potassium; Ca, calcium; P, phosphorus; Mg, magnesium; Fe, iron; Zn, zinc; Cu, copper.

† RE [%] is calculated as $\frac{\text{ICP-MS measurement} - \text{standard method measurement}}{\text{standard method measurement}} \times 100$.

‡ LLoA is the mean RE − 1.96 × standard deviation of the RE, and ULoA is the mean RE + 1.96 × standard deviation of the RE.

Supplementary Figure S1. Classification of the fertility of participants.

The Euler diagram represents the number of participants categorized by fertility classifications, including overlaps. The participants were initially classified into azoospermia and non-azoospermia groups. The latter were further classified based on semen volume, sperm concentration, and motility. Specifically, those with semen volumes <1.4 mL were categorized as having hypospermia, those with sperm concentrations <16 million/mL as having oligozoospermia, and those with motility rates <42% were categorized as having asthenozoospermia. Participants who did not meet any of these criteria were classified as having normozoospermia. Although the diagram does not reflect this, the azoospermia group included participants with unstable semen volumes, including those with volumes less than <1.4 mL.

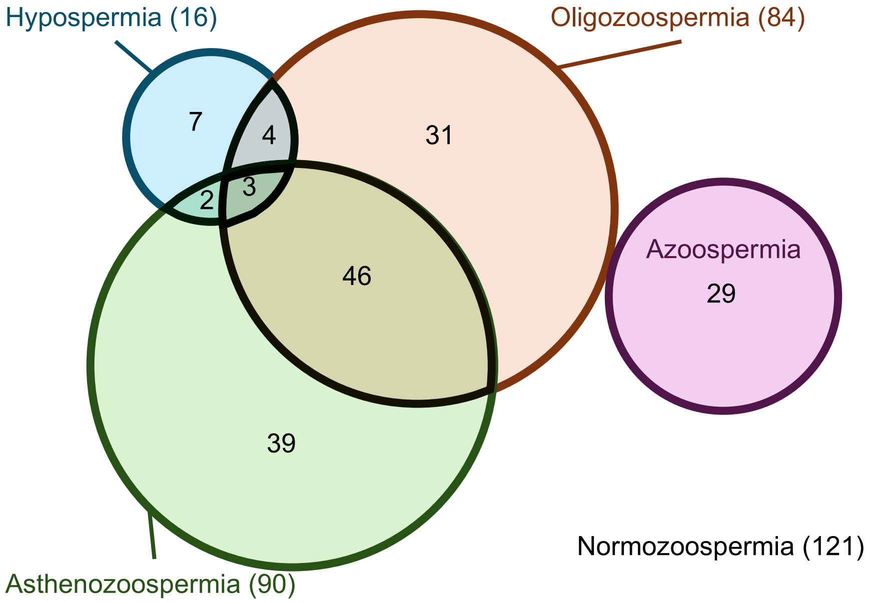

Supplementary Figure S2. Scatter plots and Passing–Bablok regression lines comparing serum mineral concentrations measured using the ICP-MS and standard methods.

The eight scatter plots represent measurements for all data (n = 282), with the vertical axis showing the ICP-MS measurements and the horizontal axis showing the standard-method measurements on a consistent scale. Owing to the lower number of significant digits in the standard method measurements compared to those for ICP-MS, the plots exhibit a discrete pattern along the horizontal axis. To maintain consistency with the other figures, sodium (Na), potassium (K), calcium (Ca), magnesium (Mg), and copper (Cu) are shown in (a), while phosphorus (P), iron (Fe), and zinc (Zn) are displayed in (b). The solid blue line represents the Passing–Bablok regression estimated from all the data, whereas the dashed blue lines indicate the upper and lower 95% confidence intervals. For Na, Ca, and P, owing to the instability of the regression equation, the confidence interval falls outside the plotting range; therefore, the dashed blue lines are partially invisible. Green-circled points represent hemolyzed samples. Red points indicate outliers due to factors other than hemolysis. These outliers have standardized residuals >3 when comparing the theoretical ICP-MS measurements, derived from the Passing–Bablok regression, with the actual ICP-MS measurements, excluding hemolyzed samples. The regression equations are displayed in the top-left corner of each plot. The dashed red line represents the identity line, where the slope is 1.00 and the intercept is 0.00. For P, the Y-axis is scaled to be 3.5 times smaller than the X-axis, and a purple dashed line, representing an identity line with a slope of 3.50 and an intercept of 0.00, is used instead of the red dashed line. For two samples of iron and three samples of zinc, the standardized residuals exceeded the upper limit of the vertical axis range and have been annotated accordingly. ICP-MS, inductively coupled plasma mass spectrometry. ICP-MS, inductively coupled plasma mass spectrometry.

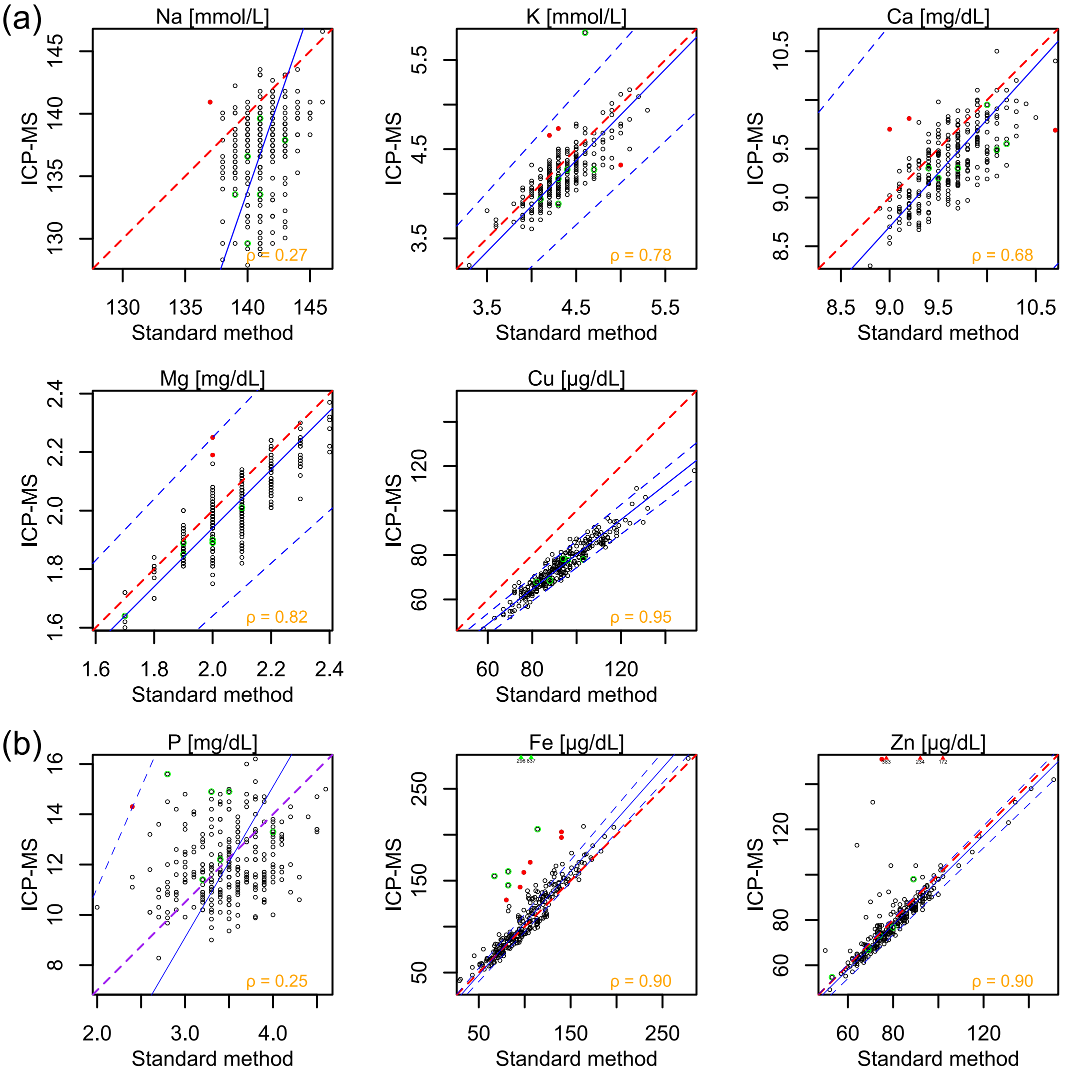

Supplementary Figure S3. Scatter plots of standardized residuals between theoretical ICP-MS measurements estimated from Passing–Bablok regression and actual ICP-MS measurements.

The eight scatter plots represent measurements from all data (n = 282), with the vertical axis indicating the standardized residuals between the theoretical ICP-MS measurements estimated using Passing–Bablok regression and the actual ICP-MS measurements, and the horizontal axis indicating the standard method measurements on a consistent scale. The standardization of residuals is calculated using the standard deviation of the residuals from non-hemolyzed samples. For elements with smaller variance in ICP-MS measurements, such as sodium (Na), potassium (K), calcium (Ca), magnesium (Mg), and copper (Cu), the vertical axis is plotted with a shorter range (a). For elements with a greater variance in ICP-MS measurements, such as phosphorus (P), iron (Fe), and zinc (Zn), the vertical axis is plotted with a longer range (b). The red dashed line represents standardized residuals with an absolute value of 3, and data points that exceed this threshold are colored red. Green circles represent hemolyzed samples, standardized using the standard deviation of residuals from non-hemolyzed samples. For one sample each of K and Fe, the standardized residuals exceeded the upper limit of the vertical axis range and are accordingly annotated.

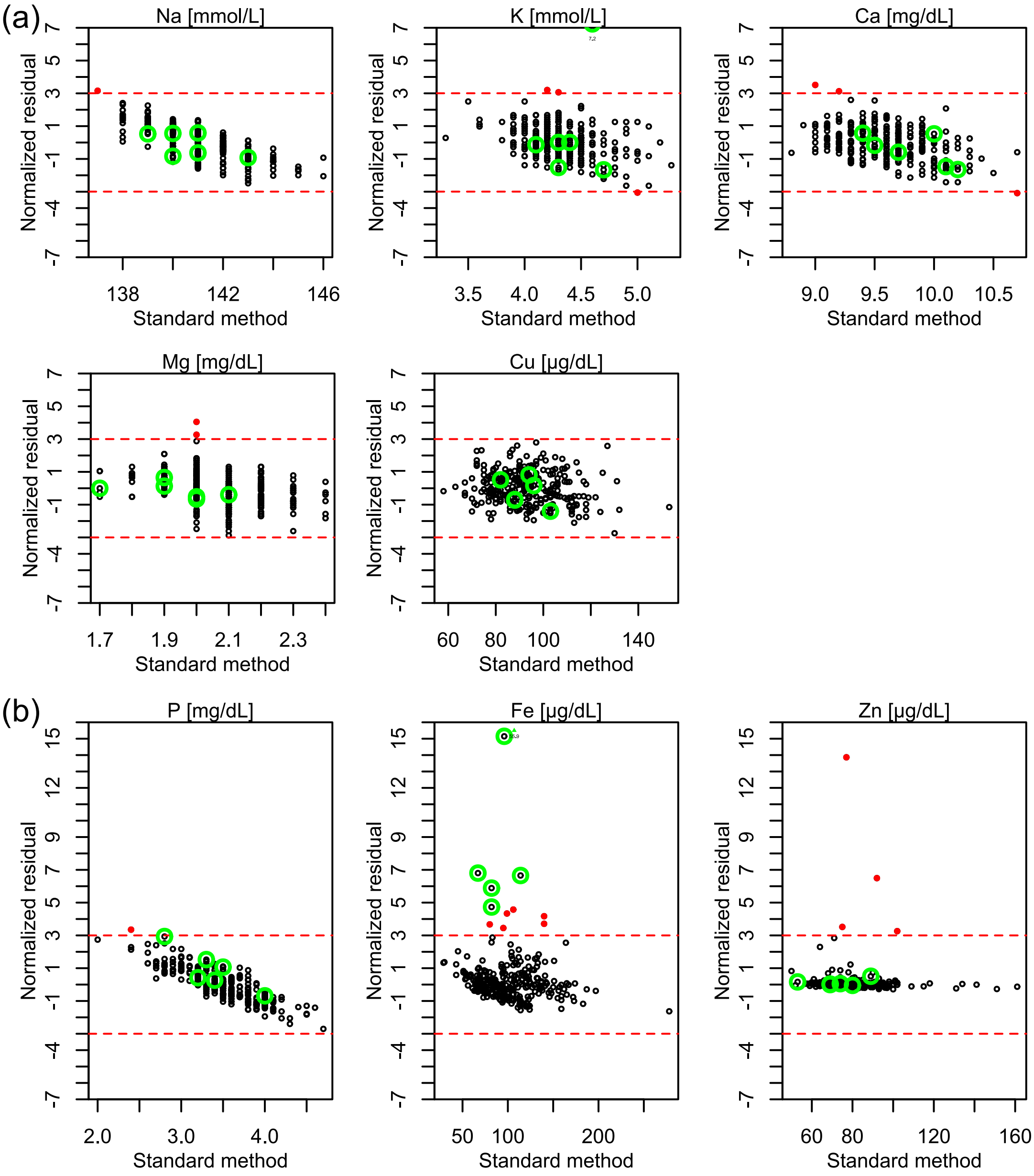

Supplement: Supplementary file 1 — Data S1. [file JCLA-39-e25140-s001.pdf]
